# Co-ordination of health care: the case of hospital emergency admissions

**DOI:** 10.1007/s10198-018-1015-x

**Published:** 2018-11-22

**Authors:** M. Kamrul Islam, Egil Kjerstad

**Affiliations:** NORCE Norwegian Research Centre AS, Bergen, Norway

**Keywords:** Incentives, Emergency bed capacity, Emergency admissions, Difference-in-differences, I10, I18, C21

## Abstract

The recognition that chronic care delivery is suboptimal has led many health authorities around the world to redesign it. In Norway, the Department of Health and Care Services implemented the Coordination Reform in January 2012. One policy instrument was to build emergency bed capacity (EBC) as an integrated part of primary care service provided by municipalities. The explicit aim was to reduce the rate of avoidable admissions to state-owned hospitals. Using five different sources of register data and a quasi-experimental framework—the “difference-in-differences” regression approach—we estimated the association between changes in EBC on changes in aggregate emergency hospital admissions for eight ambulatory care sensitive conditions (ACSC). The results show that EBC is negatively associated with changes in aggregate ACSC emergency admissions. The associations are largely consistent with alternative model specifications. We also estimated the relationship between changes in EBC on changes in each ACSC condition separately. Our results are mixed. EBC is negatively associated with emergency hospital admissions for asthma, angina and chronic obstructive pulmonary disease but not congestive heart failure and diabetes. The main implication of the study is that EBC within primary care is potentially a sensible way of redesigning chronic care.

## Introduction

The recognition that chronic care delivery is suboptimal has led many health authorities the world over to redesign chronic care. Ham [6] reviewed the international evidence on gaps in the provision of chronic care and the care that should be provided. Ham concluded that more is needed to help health care decision makers bring about the reorientation required to meet the needs of populations in which chronic diseases predominate.

For the specific context of this paper, several of Ham’s characteristics needed in a high-performing chronic care system relate—directly or indirectly—to the role of primary health care. In an effort to accomplish a reorientation of care, and with the explicit aim of reducing the number of avoidable admissions to hospital [23], the health authorities in Norway implemented the Coordination Reform in January 2012. This reform introduced three novel policy instruments based on the design of three explicit economic incentives, all involving primary health care services provided by municipalities:

(i) Forcing municipalities to internalize some of the costs of hospitalization by introducing a co-payment system, i.e. municipalities should pay 20% of the national average cost for specific diagnoses-related groups (DRGs); mainly medical rather than surgical ones. Emergency admissions were not part of the co-payment system.
(ii) Penalizing municipalities if patients with a “ready for discharge” status in need of primary care follow-up were hospitalized beyond the discharge date.
(iii) Transfers (or subsidies) to municipalities establishing 24/7 emergency bed capacity (EBC) within their primary care facilities.

The municipal co-payment scheme (i) was abolished in early 2015, with the main arguments being that the co-payment scheme did not work as envisaged^1^ and that it imposed too much risk on the municipalities, many of them small. However, the penalty scheme (ii) remains in place and municipalities are obliged to reimburse hospitals NOK 4000 per day^2^ in excess of the discharge date (as determined by the hospital).

Concerning the EBC scheme (iii), by 1 January 2016, all municipalities were legally obliged to provide EBC, either alone or in co-operation with other municipalities. EBC was established partly with transfers of funds from central health authorities to the municipalities. Municipalities could determine where to implement capacity locally, whether it was connected with an already established health centre, connected to a nursing home or in a new facility. The transfer was determined by the size of the population and based on a pre-determined average cost per bed-year. Based on population size, central health authorities determined the EBC that should be provided, and the transfer followed when municipalities documented their EBC.

Our main interest here is whether EBC achieves its aim of providing complementary capacity to hospital admission: complementary in the sense that less serious cases are handled within primary care services. Due to lack of data, we are not able to address the issue of “seriousness” directly. Rather, we analyse the association between EBC and emergency hospital admission rates for a set of ambulatory care-sensitive conditions (ACSC).^3^ If such an association exists, it may indicate that some of the emergency admissions to hospitals are avoidable. Thwaites et al. [19] issue a reminder that ‘avoidable admissions’ and ‘inappropriate admissions’ are concepts that are often used inconsistently and that they are context-dependent. The authors review the literature on emergency admissions to hospital for older people in the UK and find “varying rates of in/appropriateness, inconsistent ways of defining appropriateness and a lack of focus on the possible solutions to address the problem.” In our opinion, ACSC lend themselves well for analyses of EBC and avoidable hospitalization. Emergency admissions to hospitals related to ACSC are increasingly accepted as a meaningful indicator of primary care–specialist care co-ordination or lack of it. ACSC develop over relatively long periods of time. Timely and effective self-care, primary care or outpatient care can largely avoid the risk of crisis leading to emergency hospital admission [16].

There is evidence that patient characteristics, satisfaction with primary care/quality of primary care, and national differences are associated with differing rates of emergency hospital admissions for ACSC [7, 8, 10, 11, 15]. For example, there seems to be an inverse relation between socio-economic status and emergency admissions for ACSC. In addition, US studies show that African Americans and Hispanics have significantly higher rates of hospitalization for ACSC than do whites. Among studies focusing on the institutional aspects of care delivery, Rizza et al. [14], in an Italian study, showed the need to develop and implement effective interventions to improve the delivery of health care at the community level. They found a negative relationship between the use of community services and satisfaction with primary care, and the likelihood of experiencing ACSC-related hospitalization. In the US, Probst et al. [12] observed lower rates of admissions in areas with community health centres and rural health clinics.

There are several theoretical models that suggest that the quality of primary health care can be characterized by the presence of certain attributes. The attributes are summarized into four main primary care domains: first care contact [i.e. the general practitioner (GP) in the Norwegian health care system], longitudinality, comprehensiveness and co-ordination (e.g. [18]). There are several empirical studies of the association between avoidable hospitalization and primary care. In a recent systematic review of avoidable hospitalization and the relationship between organizational aspects of primary care, van Loenen and colleagues [21] concluded that supply of an adequate primary care physician (GP in our case) and long-term relationships between primary care physicians and patients reduces hospitalizations for chronic ACSC. The review results also acknowledged that other organizational features, such as practice type, size and specific services show mixed results or are not associated with reduced rates of hospitalization. For example, of the total 49 studies, 10 investigated the role of primary care practice organization and found that higher workloads for GPs, as well as a larger number of full-time equivalent physicians in the practice, as a measure of practice size, are factors associated with higher rates of ACSC hospitalization. Mixed results are reported for practice type, list size and access to ancillary or support services.

Other studies examine trends, geographic variations and costs associated with ACSC [3, 4, 5, 9, 22]. For example, the principal results from UK studies were that admission rates are increasing over time but with notable variations by age group and individual condition, and that admission for ACSC represents a large and increasing proportion of health care costs. Weeks et al. [22] compared France with several other European countries along with Singapore, Australia, Canada, the US and Brazil. They concluded that France has higher admission rates than most other countries, with the possible exception of the US, Australia and Brazil.

Our study examines EBC, which in principle provides a new tool for primary care services that deal with ACSC. We believe our study contributes to the existing literature in several ways. The main research question is whether EBC in primary care is associated with the reduction in the rate of emergency hospital admissions that policymakers expect, keeping in mind that the intention of EBC is not to substitute for advanced hospital emergency services but rather to complement hospital capacity, taking care of the less serious cases. We applied a quasi-experimental research design, performing several tests of robustness along with a detailed discussion of methodology. We then analysed our results and, finally, present potential policy implications. Our analyses used unique data that draws on five different sources of register data over the period 2010–2013. Municipalities took advantage of the transfers from the state at different points in time, which subsequently resulted in varying timing of the availability of emergency beds locally. We used a difference-in-differences (DID) regression approach to study the association between EBC in primary care and emergency hospital admissions for eight ACSC chronic conditions: asthma, angina, chronic obstructive pulmonary disease (COPD), diabetes (uncomplicated), congestive heart failure, atrial fibrillation, epilepsy and ulcers.

Our main finding is that changes in EBC are negatively and significantly associated with ACSC-related emergency hospital admissions. While the association is consistent across alternative model specifications, our disease-specific analyses results are mixed. For example, EBC is associated with lower rates of emergency hospital admissions for angina, asthma and COPD but not for congestive heart failure and diabetes.

The remainder of the paper is organized as follows. “Institutional contexts” gives a short presentation of important institutional features of the Norwegian primary care and secondary care financing and the division of labour between primary care and secondary care. “Estimation strategies” presents our estimation strategy and the quasi-experimental framework in the form of the DID regression methodology. “Data and variables” details the data sources and variables used in the analysis. “Results” provides the empirical results and the last section concludes the paper.

## Institutional contexts

The Norwegian health authorities conceded that co-ordination between primary care providers and hospitals was suboptimal, particularly in relation to the needs of chronically ill patients, for whom primary care can serve as a substitute for hospital care [23]. By implementing the Coordination Reform in January 2012, the health authorities in Norway took a markedly different strategy than many others in terms of attempting to reduce the number of unnecessary hospital admissions.

Before the reform, municipalities had only weak incentives to avoid hospital admissions in general, because hospital admissions were (and still are) free of charge from the perspective of primary care services. Hospitals in Norway, of which there are approximately 60 across the country, are state-owned, and hospitalization shifts costs from municipalities to central government. Hospitals are organized and run by four regional health enterprises, being the extended arms of the central health authority. Obviously, hospitals vary in scope and size across the country but are reimbursed through a “mixed” prospective payment system: prospective fixed budgets (block grants) in combination with prospective variable DRG-based remuneration. The mix of grants and DRG-based remuneration varies, determined by the national parliament on a yearly basis. At present, the mix is 50:50 grants and DRG-based remuneration.

Municipalities, of which there are approximately 430 across Norway, provide primary care services, including home care services, short- and long-term nursing home services and GP services. The two major forms of home care services are assistance with daily activities and services provided by nurses and auxiliary nurses. People with chronic conditions are in principle followed up by home care services and GPs under contract with the municipalities. Thus, to the extent that municipalities use hospitals as a buffer in terms of bed capacity and/or buffers in relation to staff deficiencies regarding home services and/or short- and long-term institutional care, the management of chronic disease unlikely to be cost-effective.

## Estimation strategies

**Fig. 1:**
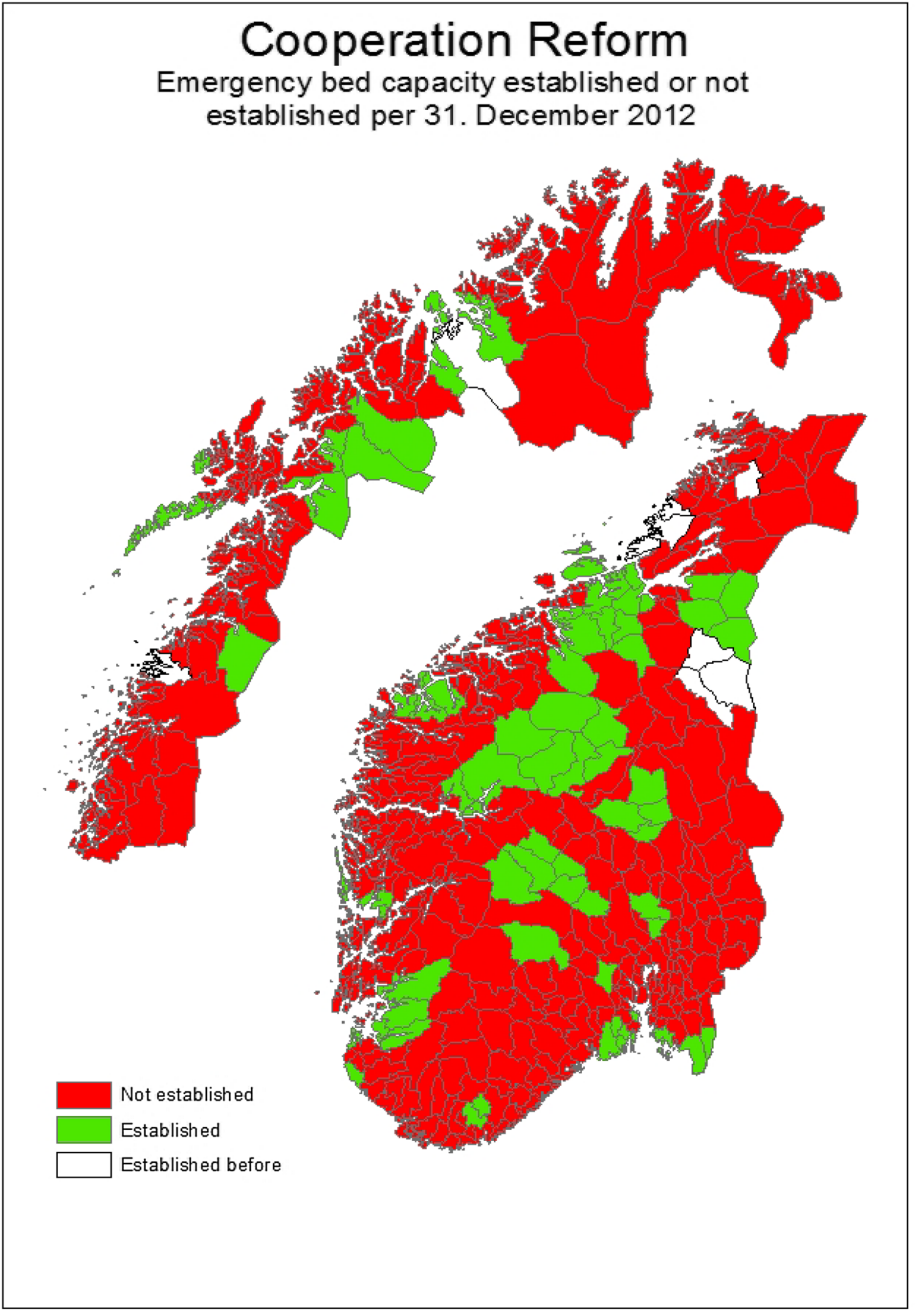
Status of the municipalities in respect to EBC established or not

Municipalities established EBC at different points in time (see Fig. 1). As an explorative estimation strategy, we used this heterogeneity in timing of the implementation of EBC as an identifying restriction, i.e. assuming that the expected change in outcomes for the non-EBC group of municipalities (control group) would be the same as it would have been for the EBC (intervention group) in the absence of EBC. The EBC group of municipalities had obvious EBC capacity available in the second period, but not in the first period, whereas the non-EBC group had EBC in neither period. We observed the same units within the groups in each period and then subtracted the expected gain (i.e. reduction in ACSC emergency admissions) in the control group from the average gain in the EBC group.

Using a quasi-experimental framework (the DID approach) we observed ACSC emergency admissions at hospitals for the two groups over two periods and estimated the association between changes in EBC in primary care and ACSC-related hospital admissions.

As shown in Fig. 1, it appears that 73 municipalities (i.e. the intervention group) took advantage of the subsidies by 31 December 2012. The remaining 294 municipalities constituted the control group.^4^ Formally, we estimate the following equation:

1 $${\text{Em}}\_{\text{ACS}}{{\text{C}}_{ijt}}={\beta _1}{R_t}+{\beta _2}{T_j}+{\beta _3}{R_t} \times {T_j}+\delta {G_{ijt}}+\gamma {S_{ijt}}+{\eta _{\text{d}}}+{e_{ijt}}.$$

The dependent variable ${\text{Em}}\_{\text{ACS}}{{\text{C}}_{ijt}}$ indicates aggregate (for all eight diagnoses) ACSC-related hospital emergency admissions by patients living in municipality *j* divided by the aggregate number of patients on the *i*th GP’s list in municipality *j* in year *t*, multiplied by 100. *R*_*t*_ and *T*_*j*_ are dummy variables indicating post-reform and intervention-group municipalities, respectively. The coefficient for ${\hat {\beta }_3}$ describes the DID estimate or impact of the reform.

To control for observable differences, we included both GP and patient-level attributes. In Eq. (1), the vector *G* includes all GP-level attributes and vector *S* comprises the average socio-economic characteristics of list patients for each GP. Given the ACSC includes pooled data across eight different diagnoses, and to control for diagnosis-specific fixed effects in Eq. (1), we included diagnoses-determined dummy variables, ${\eta _{\text{d}}}$.

Suppose the introduction of EBC is not random but systematic, i.e. takes place in municipalities with high or low average emergency admissions for ACSC or in periods with different average ACSC. To capture municipality differences that are constant over time, in Eq. (1), we further included municipality fixed-effects $({\nu _j})$ and differences over time that are common to all municipalities that are modelled by including the yearly fixed effect $({\mu _t})$. We estimated the following equation as our base model (model B1):

2 $${\text{Em}}\_{\text{ACS}}{{\text{C}}_{ijt}}={\beta ^{\prime}_1}{R_t}+{\beta ^{\prime}_2}{T_i}+{\beta ^{\prime}_3}{R_t} \times {T_j}+\delta ^{\prime}{G_{ijt}}+\gamma ^{\prime}{S_{ijt}}+{\eta _d}+{\nu _j}+{\mu _t}+{e_{ijt}}.$$

## Data and variables

We merged five different sources of register data. From the Norwegian Patient Registry, we extracted information on emergency hospital admissions, patient age, gender and diagnoses for the period from 2010 to 2013. Along with this, the KHUR is a public register administrated by the Norwegian Health Administration (HELFO), which is a unit within the Directorate of Health, used for settling fee-for-service payments to GPs from the National Insurance Scheme. From this register, we obtained information on the services provided by GPs. These include records for every GP service that generates a fee, and thus enabled us to observe the number of patient visits, patients and their diagnosis (i.e. ICPC code), and the mix of services provided to each patient such as medical/diagnostic tests and whether there were prolonged consultations. Most importantly, these data include patient and GP identifiers, which allowed us to merge information on services provided by GPs to individual patient and GP characteristics.

The GP attributes came from the GP database, while individual-level socio-economic conditions such as education, income, living alone, and disability status were from the Statistics Norway database. Finally, we collected data on whether and when emergency beds (EBC) were available in different municipalities from the Norwegian Directorate of Health.

### Dependent variables

We constructed nine dependent variables using the same approach. We created the main dependent variable ${\text{Em}}\_{\text{ASCS}}$ by aggregating emergency admissions across the eight ACSC groups for each GP in a given year in a given municipality. Dividing by the total number of patients on a GP’s list, we interpreted the variable as the share of the emergency admission usage of the list patients (multiplying by 100, the interpretation is the percentage of the emergency admission usage of the list patients):

$${\text{Em}}\_{\text{ASCS}}=\frac{{\text{Number of emergency admissions at hospital due to ASCSs by each GP}}}{{\text{GP's list length}}} \times 100.$$

Acknowledging the possibility of more than one emergency admission in a given year for the same patient, the variable remains a relevant policy measure because it measures emergency episodes relative to the number of patients on a list.

The ACSC included the diagnoses of angina, asthma, atrial fibrillation, congestive heart failure, COPD, diabetes (uncomplicated), epilepsy and ulcers. The respective dependent variables were:

(i) Percentage of emergency hospital admissions because of angina (Em_Angina);
(ii) Percentage of emergency hospital admissions because of asthma (Em_Asthma);
(iii) Percentage of emergency hospital admissions because of atrial fibrillation (Em_Atri);
(iv) Percentage of emergency hospital admissions because of congestive heart failure (Em_Heart failure);
(v) Percentage of emergency hospital admissions because of COPD (Em_ COPD);
(vi) Percentage of emergency hospital admissions because of diabetes (Em_ Diabetes);
(vii) Percentage of emergency hospital admissions because of epilepsy (Em_ Epilepsy);
(viii) Percentage of emergency admissions at hospital because of ulcers (Em_ Ulcer);

Table 1 details the variable names and their definitions.

**Table 1:** Definition of the variable

Variable name	Definition of the variable
*Dependent variables*	
Em_ASCS	$=\frac{{{\text{Number of emergency admissions at hospital due to ASCS by each GP}}}}{{{\text{GP's list length}}}} \times 100$
Em_Angina	$=\frac{{{\text{Number of emergency admissions at hospital due to angina by each GP}}}}{{{\text{GP's list length}}}} \times 100$
Em_Asthma	$=\frac{{{\text{Number of emergency admissions at hospital due to asthma by each GP}}}}{{{\text{GP's list length}}}} \times 100$
Em_Diabetes	$=\frac{{{\text{Number of emergency admissions at hospital due to diabetes without complications by each GP}}}}{{{\text{GP's list length}}}} \times 100$
Em_COPD	$=\frac{{{\text{Number of emergency admissions at hospital due to COPD by each GP}}}}{{{\text{GP's list length}}}} \times 100$
Em_Heart failure	$=\frac{{{\text{Number of emergency admissions at hospital due to heart failure by each GP}}}}{{{\text{GP's list length}}}} \times 100$
Em_Atrial	$=\frac{{{\text{Number of emergency admissions at hospital due to atrial fibrillation by each GP}}}}{{{\text{GP's list length}}}} \times 100$
Em_Epilepsy	$=\frac{{{\text{Number of emergency admissions at hospital due to epilepsy by each GP}}}}{{{\text{GP's list length}}}} \times 100$
Em_Ulcer	$=\frac{{{\text{Number of emergency admissions at hospital due to ulcer by each GP}}}}{{{\text{GP's list length}}}} \times 100$
*Independent variables*	
Norw_GP	Whether GP comes from Norway = 1, otherwise = 0
Male_GP	Whether GP is a male = 1, female = 0
Specialist	Whether GP is a specialist = 1; otherwise = 0
Age_GP	GP’s age in year
Consult_GP	Number of GP’s consultation
Share_LC_GP	Share of patient per GP with long consultation
Share_Test_GP	Share of patient per GP done with medical/diagnostic test
Visist_GP	Number of patient visit per GP
Pat_Age	Patient average age per GP
Pat_Mal	Share of male patient per GP
Pat_Edu1	Share of patient per GP with elementary level of education
Pat_Alone	Share of patient per GP with live alone
Pat_Disable	Share of patient per GP with disability
Pat_Wage	Average early wage income per GP divided by 1000
Angina	If angina = 1
Asthma	If asthma = 1
Diabetes	If diabetes without complications = 1
COPD	If COPD = 1
Heart	If heart failure = 1
Atrial fibrillation	If atrial fibrillation = 1
Epilepsy	If epilepsy = 1
Ulcer	If ulcer = = 1

### Control variables

#### GP attributes

As mentioned above, van Loenen et al. [21] reviewed empirical studies of ACSC-related hospitalization and their relationship with different primary care organizational aspects. The review identifies different attributes used to describe primary care services with proxies for workload among them. The GP attributes included the following proxies for workload: number of consultations, share of long consultations, share of patients per GP with registered medical or diagnostic tests and number of patient visits per calendar year. Our dependent variables were constructed at GP-level; therefore, we further controlled for characteristics of the GP: country of birth, gender, age and whether the GP was a specialist or not.

#### Patient attributes averaged by GP

The patient attributes we included as control variables in the analyses were age, gender and socio-economic characteristics, including education, marital status, disability status and wage income. As all analyses were at GP-level; we averaged these patient attributes for each GP list. As presented in the introduction, studies of hospitalization rates for ACSC (e.g., [7, 8, 10, 11, 15]) show that there is an inverse relation between socio-economic status and emergency admissions for ACSC.

## Results

### Descriptive statistics

From 2010 and onwards, there has been a downward trend in the rate of emergency admissions in aggregate terms (Em_ASCS), from 0.95 percentage points in 2010 to 0.91 percentage points in 2013 (Table 2): a reduction of approximately half a percentage point overall. A downward trend also holds for some of the disease-specific rates. However, the COPD rate has increased over time (from 0.165 to 0.176%), while for ulcers and atrial fibrillation, the rates are stable. The independent variables also appear to be rather stable over time, as shown in Table 2.

**Table 2:** Descriptive statistics for the variables used in the analyses: 2010–2013

Variable	2010 (*N* = 24,130)		2011 (*N* = 24,522)		2012 (*N* = 24,961)		2013 (*N* = 25,363)	
	Mean (%)	SD (%)	Mean (%)	SD (%)	Mean (%)	SD (%)	Mean (%)	SD (%)
Em_ASCS	0.950	0.520	0.896	0.564	0.924	0.586	0.915	0.591
Em_Angina	0.178	0.186	0.160	0.180	0.156	0.197	0.152	0.195
Em_Asthma	0.062	0.098	0.056	0.093	0.050	0.100	0.048	0.085
Em_COPD	0.167	0.221	0.174	0.252	0.178	0.234	0.179	0.243
Em_Heart failure	0.179	0.187	0.162	0.189	0.168	0.183	0.166	0.175
Em_Diabetic	0.040	0.067	0.035	0.062	0.035	0.069	0.035	0.069
Em_Ulcer	0.007	0.025	0.006	0.023	0.007	0.026	0.006	0.025
Em_Atrial	0.234	0.207	0.221	0.204	0.246	0.231	0.250	0.241
Em_Epilepsy	0.084	0.142	0.082	0.136	0.086	0.138	0.080	0.122
Norw_GP	0.714	0.452	0.704	0.457	0.695	0.460	0.697	0.460
Male_GP	0.675	0.469	0.665	0.472	0.653	0.476	0.645	0.479
Specialist	0.660	0.474	0.663	0.473	0.668	0.471	0.666	0.471
Age_GP	49.90	10.03	50.00	10.23	49.96	10.42	49.91	10.58
Consult_GP	3.422	1.288	3.367	1.302	3.366	1.156	3.334	1.112
Share_LC_GP	0.353	0.186	0.349	0.182	0.362	0.184	0.376	0.186
Share_Test_GP	0.575	0.144	0.586	0.140	0.591	0.139	0.589	0.139
Visist_GP	10.56	10.19	10.47	10.25	10.21	9.975	9.898	9.413
Pat_Age	51.43	24.80	51.39	25.11	51.69	24.67	52.58	24.80
Pat_Mal	0.451	0.310	0.447	0.316	0.447	0.318	0.448	0.320
Pat_Edu1	0.341	0.113	0.329	0.108	0.325	0.107	0.317	0.107
Pat_Alone	0.308	0.092	0.304	0.087	0.305	0.086	0.307	0.087
Pat_Disable	0.158	0.071	0.154	0.067	0.150	0.066	0.158	0.070
Pat_Wage	145.7	55.04	153.5	55.06	162.1	60.28	158.6	60.67
Angina	0.135	0.342	0.132	0.339	0.132	0.338	0.131	0.337
Astma	0.141	0.348	0.141	0.348	0.141	0.348	0.141	0.348
Kols	0.138	0.345	0.139	0.346	0.139	0.345	0.139	0.346
KrHjSvikt	0.130	0.336	0.132	0.338	0.131	0.338	0.130	0.337
Diabets_NC	0.144	0.351	0.145	0.352	0.145	0.352	0.146	0.353
Ulcer	0.028	0.164	0.026	0.160	0.027	0.162	0.029	0.167
Atrial_F	0.144	0.351	0.146	0.353	0.144	0.352	0.145	0.352
Epilepsy	0.140	0.347	0.140	0.347	0.141	0.349	0.140	0.347

**Fig. 2:**
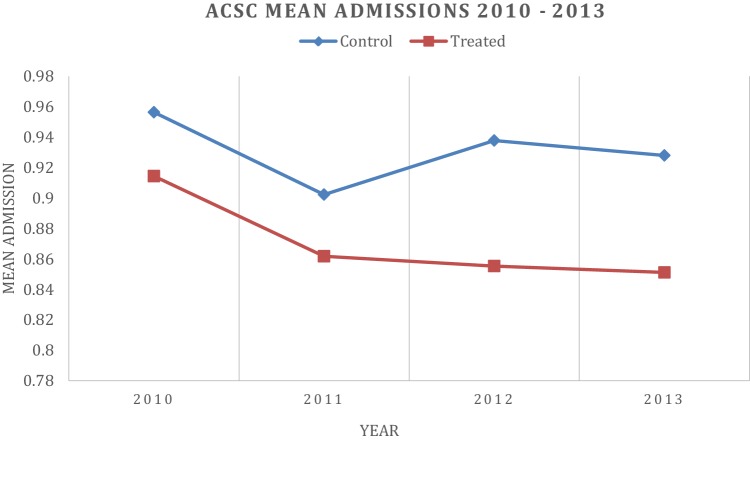
ACSCs hospital admissions over the year for control and treatment municipality

**Fig. 3:**
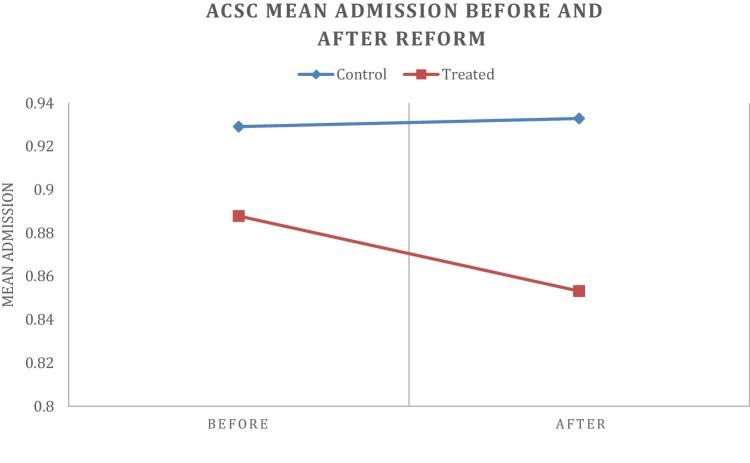
ACSCs hospital admissions before and after reform in control and treatment municipality

**Fig. 4:**
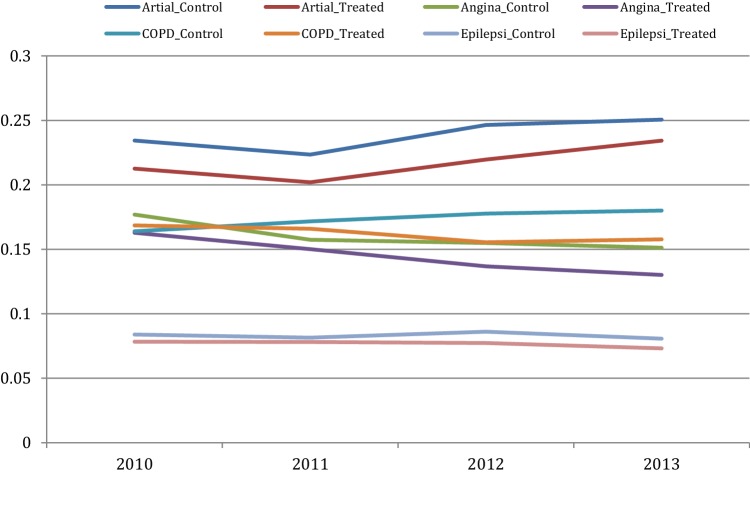
Atrial F, angina, COPD and epilepsy emergency hospital admissions over the year for control municipality (control) and treatment municipality

**Fig. 5:**
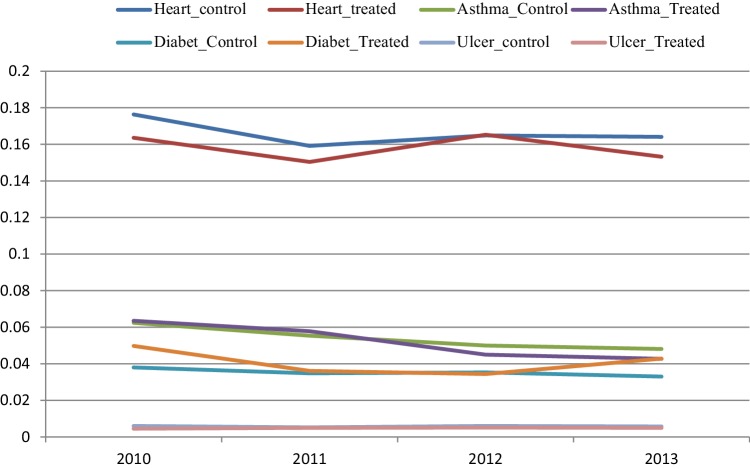
Heart failure, asthma, diabetes and ulcer emergency hospital admissions over the year for control municipality (control) and treatment municipality

Figures 2, 3, 4 and 5 depict the aggregate trends for the control or non-EBC group of municipalities and the EBC group, and the same for the disease-specific trends. The trend for Em_ASCS is downward for both groups of municipalities. There are some differences in the disease-specific trends, notably for angina, asthma and COPD. For these conditions, the reduction in admission rates is greater in municipalities that have established EBC compared to those which have not.

Table 3 provides the aggregate averages of EM_ACSC and for the disease-specific cases. With few exceptions, the average level is highest for the control group in the pre-reform period. More specifically, the yearly average of EM_ACSC for the control group in the pre-reform period (2010–2011) is almost equal (at the second decimal place, 0.929–0.933) to the post-reform period (2012–2013). The EBC group of municipalities experiences a reduction in Em_ASCS (from 0.888 percentage points to 0.853 percentage points) of approximately half a percent.

**Table 3:** Descriptive statistics of the dependent variables for control and treatment municipalities: before and after reform (with alternative definitions)

Variable	Before reform (2010–2011)		After reform			
			2012–2013		2013 only	
	Control	Treatment	Control	Treatment	Control	Treatment
	Mean (SD)	Mean (SD)	Mean (SD)	Mean (SD)	Mean (SD)	Mean (SD)
Em_ACSC	0.929 (0.552)	0.888 (0.496)	0.933 (0.601)	0.853 (0.517)	0.928 (0.608)	0.851 (0.492)
Em_Angina	0.170 (0.187)	0.160 (0.167)	0.157 (0.200)	0.136 (0.170)	0.155 (0.202)	0.133 (0.151)
Em_Asthma	0.059 (0.095)	0.061 (0.097)	0.049 (0.094)	0.044 (0.087)	0.048 (0.084)	0.043 (0.092)
Em_COPD	0.171 (0.241)	0.168 (0.216)	0.182 (0.243)	0.159 (0.215)	0.183 (0.251)	0.160 (0.197)
Em_Heart failure	0.172 (0.192)	0.161 (0.168)	0.168 (0.177)	0.163 (0.190)	0.168 (0.176)	0.156 (0.172)
Em_Diabetic	0.037 (0.064)	0.042 (0.067)	0.034 (0.070)	0.039 (0.064)	0.033 (0.069)	0.043 (0.065)
Em_Ulcer	0.006 (0.024)	0.005 (0.023)	0.007 (0.025)	0.006 (0.028)	0.006 (0.024)	0.005 (0.028)
Em_Atrial	0.230 (0.207)	0.210 (0.199)	0.251 (0.241)	0.230 (0.212)	0.253 (0.245)	0.237 (0.220)
Em_Epilepsy	0.084 (0.141)	0.079 (0.128)	0.084 (0.134)	0.076 (0.108)	0.081 (0.125)	0.074 (0.108)

There are some notable differences in the disease-specific averages. In the case of COPD (Em_COPD) in particular, the average is *higher* post-reform compared with the pre-reform average (0.168 versus 0.179, an increase of more than half a percentage point). For the EBC group, the result is the opposite: a reduction of 0.01 percentage point (from an average of 0.167 to an average of 0.157). For diabetes (Em_Diabetes), the pre-reform average is highest for the EBC group and does not quite catch up to the post-reform level (0.34 versus 0.35) although the reduction measured in percentage points is clearly in favour of the EBC group (0.002 versus 0.008).

Analogous to Tables 3 and 4 illustrates descriptive statistics of the independent variables before and after reform, and for the control and intervention municipalities groups. As seen in Table 4, the mean values of different GP-level covariates and patient-level characteristics are very similar between control and intervention municipalities in the pre-reform period. This similar trend is also observed in the post-reform period. For example, the average age of a GP is around 50 years and average age of the patient per GP is around 52 years in all groups. Similarly, the share of GPs with specialization is around 66% and the mean number of consultations per list patient per year is around 3. During the pre-reform period, the average number of patient visits per GP per year is of similar magnitude: 10.55 for control municipalities and 10.31 for intervention municipalities.

**Table 4:** Descriptive statistics of the covariates for control and treatment municipalities: before and after reform (with alternative definitions)

Variable	Before reform (2010–2011)		After reform			
			2012–2013		2013 only	
	Control	Treatment	Control	Treatment	Control	Treatment
	Mean (SD)	Mean (SD)	Mean (SD)	Mean (SD)	Mean (SD)	Mean (SD)
Norw_GP	0.710 (0.454)	0.705 (0.456)	0.696 (0.460)	0.696 (0.460)	0.698 (0.459)	0.689 (0.463)
Male_GP	0.668 (0.471)	0.681 (0.466)	0.649 (0.477)	0.646 (0.478)	0.646 (0.478)	0.637 (0.481)
Specialist	0.661 (0.474)	0.665 (0.472)	0.671 (0.470)	0.645 (0.478)	0.672 (0.470)	0.640 (0.480)
Age_GP	50.02 (10.06)	49.60 (10.49)	50.10 (10.47)	49.13 (10.64)	50.10 (10.56)	48.98 (10.64)
Consult_GP	3.386 (1.181)	3.436 (1.770)	3.344 (1.128)	3.383 (1.161)	3.334 (1.125)	3.338 (1.046)
Share_LC_GP	0.355 (0.184)	0.329 (0.182)	0.373 (0.185)	0.351 (0.183)	0.380 (0.186)	0.356 (0.181)
Share_Test_GP	0.578 (0.144)	0.594 (0.131)	0.589 (0.140)	0.596 (0.131)	0.588 (0.141)	0.593 (0.129)
Visist_GP	10.55 (10.15)	10.31 (10.60)	10.07 (9.594)	9.979 (10.20)	9.908 (9.334)	9.846 (9.801)
Pat_Age	51.32 (24.94)	51.84 (25.07)	52.18 (24.64)	51.95 (25.24)	52.65 (24.76)	52.24 (25.01)
Pat_Mal	0.451 (0.313)	0.437 (0.313)	0.449 (0.319)	0.443 (0.3199)	0.449 (0.319)	0.445 (0.322)
Pat_Edu1	0.333 (0.113)	0.346 (0.092)	0.319 (0.110)	0.332 (0.090)	0.315 (0.110)	0.324 (0.087)
Pat_Alone	0.308 (0.092)	0.295 (0.073)	0.309 (0.089)	0.292 (0.073)	0.310 (0.088)	0.294 (0.078)
Pat_Disable	0.155 (0.070)	0.162 (0.063)	0.153 (0.068)	0.162 (0.070)	0.157 (0.070)	0.166 (0.071)
Pat_Wage	151.6 (55.76)	139.6 (51.02)	162.0 (60.55)	151.9 (59.54)	160.1 (60.63)	150.9 (60.31)
Angina	0.134 (0.341)	0.132 (0.339)	0.131 (0.337)	0.134 (0.341)	0.131 (0.337)	0.131 (0.337)
Asthma	0.141 (0.348)	0.141 (0.348)	0.141 (0.348)	0.138 (0.345)	0.141 (0.348)	0.140 (0.347)
Kols	0.139 (0.346)	0.137 (0.344)	0.139 (0.346)	0.136 (0.343)	0.139 (0.346)	0.138 (0.345)
KrHjSvikt	0.129 (0.336)	0.139 (0.346)	0.131 (0.337)	0.130 (0.336)	0.130 (0.337)	0.129 (0.335)
Diabetes_NC	0.144 (0.351)	0.144 (0.351)	0.145 (0.352)	0.149 (0.356)	0.144 (0.352)	0.154 (0.361)
Ulcer	0.028 (0.164)	0.024 (0.153)	0.028 (0.166)	0.025 (0.156)	0.029 (0.169)	0.026 (0.159)
Atrial_F	0.145 (0.352)	0.144 (0.351)	0.145 (0.352)	0.145 (0.352)	0.145 (0.352)	0.146 (0.353)
Epilepsy	0.140 (0.347)	0.138 (0.345)	0.140 (0.347)	0.142 (0.349)	0.140 (0.347)	0.136 (0.343)

### Difference-in-differences (DID) estimates

Table 5 reports the results of the DID estimates of the effects of EBC on aggregate emergency ACSC admissions for alternative models. Our DID estimates of the base model suggest a reduction of ACSC in aggregate by 0.03 percentage points (Table 5). In other words, ACSC fell by about 0.38 per GP (with an average of 1223 listed patients per GP). Based on the overall sample average of ACSC before the reform (0.922), introducing EBC reduced emergency admissions related to ACSC by around 3.4% (i.e. $\frac{{0.0314}}{{0.9220}}$ × 100).

**Table 5:** Effect of the emergency bed capacity within primary care on ACSC hospital admission: difference-in-differences estimates with alternative combinations of before and after reform years (cluster standard errors in municipalities are in the parentheses)

	Model B1 Reform = 1 (year = 2012 and 2013) Reform = 0 (year = 2010 and 2011)	Model B2 Reform = 1 (year = 2013) Reform = 0 (year 2010 and 2011)	Model S1 Reform = 1 (year 2012–2013) Reform = 0 (year = 2010 and 2011)	Model S2 Reform = 1 (year = 2013) Reform = 0 (year 2010 and 2011)
Reform	0.0024 (0.0044)	− 0.0073 (0.0118)	0.0016 (0.0104)	− 0.0112 (0.0111)
Treat^a^	0.7419*** (0.0211)	− 0.1363*** (0.0240)	0.1612*** (0.0203)	0.1392*** (0.0209)
Reform × treat	− 0.0314** (0.0190)	− 0.0281* (0.0199)	− 0.0331 (0.0330)	− 0.0281* (0.0212)
*Control variables*				
Norw_GP	0.0402*** (0.0126)	0.0415*** (0.0153)	0.0401*** (0.0126)	0.0415*** (0.0153)
Male_GP	0.1432*** (0.0118)	0.1557*** (0.0117)	0.1432*** (0.0118)	0.1557*** (0.0117)
Specialist	− 0.0297** (0.0136)	− 0.0232* (0.0139)	− 0.0296** (0.0136)	− 0.0232* (0.0139)
Age_GP	0.0033*** (0.0006)	0.0030*** (0.0007)	0.0033*** (0.0006)	0.0030*** (0.0007)
Consult_GP	− 0.0034 (0.0041)	− 0.0024 (0.0045)	− 0.0034** (0.0041)	− 0.0023 (0.0045)
Share_LC_GP	− 0.0090 (0.0306)	0.0164 (0.0298)	− 0.0090 (0.0306)	0.0164 (0.0298)
Share_Test_GP	0.1414*** (0.0391)	0.1285*** (0.0426)	0.1418*** (0.0390)	0.1289*** (0.0426)
Visist_GP	0.0084*** (0.0005)	0.0084*** (0.0005)	0.0084*** (0.0005)	0.0084*** (0.0005)
Pat_Age	0.0017*** (0.0002)	0.0017*** (0.0002)	0.0017*** (0.0002)	0.0017*** (0.0002)
Pat_Male	0.0131** (0.0067)	0.0104 (0.0067)	0.0130** (0.0066)	0.0103 (0.0067)
Pat_Edu1	0.0679 (0.0848)	0.0701 (0.0836)	0.0681 (0.0848)	0.0707 (0.0836)
Pat_Alone	0.0723 (0.0802)	0.0606 (0.0762)	0.0729 (0.0802)	0.0611 (0.0762)
Pat_Disable	− 0.0917 (0.0977)	− 0.0710 (0.1010)	− 0.0927*** (0.0299)	− 0.0716 (0.1010)
Pat_Wage	− 0.0001*** (0.0000)	− 0.0014*** (0.0001)	− 0.0014*** (0.0001)	− 0.0014*** (0.0001)
Number of observation	98,976	74,015	98,976	74,015
*R*-squared	0.36	0.36	0.36	0.36
Year fixed-effects	Yes	Yes	Yes	Yes
Municipality fixed-effects	Yes	Yes	Yes	Yes

In models S1 and S2: Treat = 1 if municipality implemented emergency bed by June 30, 2012. DID coefficients (i.e. Reform × Treat) tested against one-sided alternatives

‘*’, ‘**’ and ‘***’ represents significance level at the 10%, 5% and 1% level respectively

^a^In models B1 and B2: Treat = 1 if municipality implemented emergency bed by December 31, 2012

Notice that for all models, we estimated clustered standard errors to allow for arbitrary within-group correlations at municipality level. We hypothesized that the impact of EBC on ASCS admissions would be negative, so the appropriate alternative hypothesis was a non-negative coefficient. Thus, in each model we tested the hypothesis against the one-sided alternative.

Table 5 also shows that most of the control variables were significant. All other things being equal, we found a positive effect on emergency ACSC admissions for GP characteristics including being a native Norwegian, male and older GP and negative effects for specialized GPs and those GPs providing a larger number of consultations. Patient characteristics (averaged at the GP level) also exhibit a significant relation with emergency admissions for ACSC. In particular, higher patient age and a larger share of male and less educated patients, and those living alone tend to increase emergency admissions. Conversely, larger average wage income per listed patient tends to decrease emergency admissions.

### Robustness check

To study the robustness of our results, we applied two categories of analysis. In the first category, we estimated three alternative models using Eq. (2). The models tested the sensitivity of our baseline DID estimate and they differ by:

(i) Alternative definition of the post-reform period (i.e. post-reform year only includes 2013)
(ii) Alternative definition of intervention municipalities group based on when municipalities introduced EBC locally (i.e. intervention group includes municipalities by the end of June 2012)
(iii) Combinations of (i) and (ii).

#### Alternative definition of the post-reform period (i): Model B2

Our base model (model B1) specifies the post-reform period (i.e. *R*_*t*_ = 1) as 2012 and 2013, and 2010 and 2011 as the pre-reform period (i.e. *R*_*t*_ = 0). The EBC group consists of municipalities that introduced EBC by 31 December 2012 (i.e. *T* = 1). A lag effect of the reform on ACSC admissions is quite likely viable, and this alternative characterization of the post-reform period may elicit such an effect, if any. Considering this aspect of the robustness of our base model, Model B2 entails *the same definition of the EBC group* and the *same pre-reform period* but with a *different construction of the post-reform period*, i.e. the post-reform dummy includes only observations for the year 2013. Thus, corresponding to Eq. (2), model B2 differs only in *R*_*t*_ = 1 if the post-reform period = 2013 (*n* = 74,105). The corresponding estimated coefficient of the interaction term Reform_1_ × Treat $(\beta _{3}^{{{\text{B}}2}})$ describes the DID estimate of the reform for this alternative post-reform period.

#### Alternative definition of intervention municipalities group (ii): model S1

Our second robustness check, model S1, applies the same definition for the post-and pre-reform periods as our base model B1 but with a different definition of the EBC group of municipalities (intervention municipalities). In this specification, the EBC group consists of municipalities that implemented EBC by 30 June 2012. In model B1, *31 December 2012 is applied*. In other words, in model S1, *T*_*i*_ = 1 if the EBC group consists of municipalities that implemented EBC by the end of June 2012 (with this definition, the number of municipalities in the *treatment group* is 12) and the corresponding coefficient Reform × Treat_1_$(\beta _{3}^{{{\text{S}}1}})$ describes the DID estimate for this model.

#### Alternative definition of intervention municipalities and post-reform period (iii): model S2

The third robustness check is by model S2, where we use an identical definition of the treatment municipalities as in model S1 (i.e. *T*_*i*_ = 1 if the EBC group consists of municipalities that implemented EBC by the end of June 2012) but with a different definition of the post-reform period compared to Model B2. Here *R*_*t*_ = 1 includes only post-reform observations for 2013. In this model, the corresponding estimated coefficient Reform_1_ × Treat_1_$(\beta _{3}^{{{\text{S}}1}})$ describes the DID estimate.

As shown in Table 5, regardless of how we defined the post-reform period or group of intervention municipalities, the interaction term (i.e. the estimated coefficient for ${\hat {\beta }_3}$ in Eq. 2)^5^ is negative and statistically significant in three out of the four models. However, the clustered standard errors are larger for the second two models (S1 and S2). These models include comparatively fewer treatment municipalities (i.e. number of municipalities = 12) than in models B1 and B2 (number of municipalities = 73). Nonetheless, the absolute magnitudes of the coefficients are close. These results suggest that EBC had a negative effect on emergency hospital admissions relating to ACSC. Even if the effects look rather weak, the negative effect is consistent regardless of alternative definitions of the post-reform period and/or compositions of treatment municipalities, i.e. regardless of combinations of (i) and (ii).

### Robustness checks for “artificial” reform and treatment municipalities

In the second category of robustness checks, we further estimated three different models based on Eq. (2):

(i) “artificial” reform period, i.e. before = 2010 and after = 2011
(ii) “artificial” intervention municipalities
(iii) “artificial” intervention municipalities and post-reform period 2013 only.

#### “Artificial” reform period (i)

A key assumption of the DID is that of a common or parallel trend. This states in general terms that in the absence of treatment, the average outcomes of the treatment group and the control group would follow parallel paths over time. In other words, pre-reform data may indicate that the trends are identical (Angrist and Pischke [1]). Our first robustness check of the second category (i) considered this issue. Following common practice, we graphically examined average ACSC emergency admissions from 2010 to 2013 to see whether the common trend assumption is satisfied in the years before implementation of the reform in January 2012. Figure 2 depicts ACSC hospital admissions over time for both non-EBC municipalities and EBC municipalities. As shown in Fig. 2, average ACSC admissions decreased during 2010 and 2011 in both groups and the trends are generally parallel.

Moreover, defining the pre-reform period as 2010 only and the post-reform period to be 2011 is a way to “artificially” scrutinize the parallel trend assumption (i). We know of no other particular events in 2010 and 2011 that could have systematically affected ACSC emergency admissions in Norway. Thus, if this intervention has any effect, it leads us to suspect that the effects revealed in Table 5 are spurious. However, as indicated by the second column in Table 6, the DID setup including an interaction term for this “artificial” reform specification is revealed as insignificant. This result implies that municipalities and hospitals have not changed their coordinating efforts, at least not those affecting ACSC-related admissions during 2010 and 2011.

**Table 6:** Difference-in-differences estimates for “placebo” reform effect on ACSCs hospital admission: construct artificial/placebo treatment municipalities and placebo reform year (cluster standard errors in municipalities are in the parentheses)

Variable	Placebo reform effect^a^	Artificial/placebo treatment municipalities ^b^Reform = 1 (year = 2012 and 2013) Reform = 0 (year = 2010 and 2011)	Artificial/placebo treatment municipalities ^b^Reform = 1 (year = 2013) Reform = 0 (year = 2010 and 2011)
Placebo_Reform	− 0.0438*** (0.110)	–	–
Treat	− 2.743*** (0.295)	–	–
Placebo_Reform × Treat	− 0.0072 (0.0247)	–	–
Reform	–	− 0.0008 (0.0121)	–
Placebo_Treat	–	0.7305*** (0.0195)	–
Reform × Placebo_Treat	–	− 0.0040 (0.0154)	–
Reform1^c^	–	–	− 0.0137 (0.0127)
Placebo_Treat	–	–	− 1.044*** (0.0547)
Reform1 × Placebo_Treat	–	–	− 0.0037 (0.0175)
Number of observation	48,652	98,976	74,015
*R*-squared	0.34	0.36	0.36
Year fixed-effects	Yes	Yes	Yes
Municipality fixed-effects	Yes	Yes	Yes

All the models are also control for the variables included in Table 4. DID coefficients (i.e. Reform × Treat/Reform × Placebo_Treat/Reform1 × Placebo_Treat) tested against one-sided alternatives

‘*’, ‘**’ and ‘***’ represents significance level at the 10%, 5% and 1% level respectively

^a^Placebo reform = 1, if year = 2011 and Placebo reform = 0, if year = 2010

^b^The Placebo treatment municipalities are created arbitrarily/randomly using Norwegian county numbers (see the map in Appendix). For example, municipalities belongs to the first three counties (1–3) are considered to be in the treatment municipality and the next 3 (4–6) considered as control municipality, and doing the same procedure for the rest of the counties

^c^Reform1 = 1 if after Reform includes year 2013; Reform13 = 0 if before Reform includes year 2010–2011

#### “Artificial” intervention municipalities (ii)

As a further robustness check of the second category, in (ii) we created “artificial” control and intervention municipalities groups. In particular, we created the EBC municipalities group (intervention group) arbitrarily using Norwegian county numbers (see the map in Appendix). For example, municipalities belonging to the first three counties (1–3) are considered to be in the EBC group, while the next three (counties 4–6) are considered control municipalities. Similarly, counties 7–9 are considered to be in the EBC group and so forth. Using this process, we re-estimated Eq. 2. A significantly different effect for this “artificial” control and intervention municipalities groups would lead us to question whether the introduction of EBC had a real impact on ASCS admissions. However, as shown in the third column in Table 6, the interaction coefficient (i.e. DID estimate) for the redefined “artificial” EBC municipalities group is insignificant.

#### “Artificial” intervention municipalities and post-reform period 2013 only (iii)

Corresponding to Model B2 (see Table 5), in our third alternative (iii) we re-estimated the model for the “artificial” intervention municipalities groups and the alternative post-reform year specification (i.e. *R*_*t*_ = 1 if year = 2013). As shown in the fourth column in Table 6, the interaction coefficient (i.e. DID estimate) is also insignificant for this “artificial” alternative specification as well.

Overall, both the first (sensitivity analyses) and second category of the robustness checks presented in Tables 5 and 6 respectively, suggest that our main DID estimates are robust.

### Difference-in-differences (DID) estimates for each ACSC separately

Using Eq. (2), Table 7 illustrates the results of the eight different diagnoses with the alternate post-reform definitions. As shown in Table 7, except for heart failure and ulcers, EBC has a negative association with emergency admissions for the six remaining diagnoses. However, across all model specifications, we only observe a significant negative impact for two of the diagnoses: angina and COPD. The relatively small number of observations for some of the other diagnoses may be a reason for the statistical insignificance effects.

**Table 7:** Effect of the establishing emergency bed capacity within primary care on ACSCs hospital admission: difference-in-differences estimates for specific diagnoses with alternative before and after reform years (cluster standard errors in municipalities are in the parentheses)

Variable	Angina	Asthma	Diabetes	COPD	Heart failure	Atrial_F	Epilepsy	Ulcer
Reform	− 0.005 (0.005)	− 0.004* (0.002)	− 0.003** (0.002)	0.022*** (0.005)	− 0.011** (0.005)	0.004 (0.007)	0.009** (0.004)	0.000 (0.003)
Treat	− 0.158*** (0.015)	0.153*** (0.008)	0.003 (0.003)	0.226*** (0.013)	0.370** (0.017)	0.012* (0.009)	0.014 (0.006)	0.068*** (0.011)
Reform × Treat^a^	− 0.012* (0.009)	− 0.006 (0.005)	− 0.002 (0.003)	− 0.016* (0.012)	0.009 (0.008)	− 0.004 (0.010)	− 0.005 (0.006)	0.003 (0.006)
Number of observation	13,110	13,935	14,334	13,721	12,954	14,336	13,871	2715
*R*-squared	0.45	0.19	0.12	0.26	0.27	0.31	0.21	0.17
Reform1	− 0.004 (0.005)	− 0.004* (0.003)	− 0.005*** (0.002)	0.027*** (0.006)	− 0.012*** (0.005)	0.011** (0.006)	0.004 (0.004)	− 0.001 (0.003)
Treat	0.162*** (0.016)	0.155*** (0.008)	0.024 (0.003)	− 0.209*** (0.035)	0.025* (0.015)	− 0.298*** (0.029)	− 0.047*** (0.010)	0.005 (0.015)
Reform1 × Treat^b^	− 0.009 (0.011)	− 0.006 (0.007)	0.004 (0.004)	− 0.019* (0.014)	0.002 (0.008)	− 0.001 (0.010)	− 0.005 (0.006)	0.005 (0.009)
Number of observation	9816	10,427	10,724	10,263	6442	10,731	10,340	2042
*R*-squared	0.47	0.17	0.17	0.28	0.31	0.29	0.22	0.19

All the models are also control for the variables included in Table 4. DID coefficients (i.e. Reform × Treat/Reform1 × Treat) tested against one-sided alternatives

‘*’, ‘**’ and ‘***’ represents significance level at the 10%, 5% and 1% level respectively

^a^Reform = 1 if after Reform includes year 2012–2013; Reform = 0 if before Reform includes year 2010–2011. Treat = 1 if municipality implemented emergency bed by December 31, 2012

^b^Reform1 = 1 if after Reform includes year 2013; Reform13 = 0 if before Reform includes year 2010–2011

## Conclusions

Chronic care delivery is suboptimal in many countries, and policymakers around the world pursue a reduction in avoidable hospital admissions by redesigning health care delivery. In Norway, the Department of Health and Care Services implemented the Coordination Reform in January 2012. One of the tools of the reform was the introduction of EBC within primary care services with the aim of reducing emergency admissions to state-owned hospitals run by regional health enterprises. EBC is not meant to substitute for all kinds of emergency capacity provided at hospitals, but rather as a complementary capacity taking care of the less serious emergency cases. In the context of ACSC, the ability to handle at least some of the emergency cases outside hospitals would release the pressure on hospitals.

Our paper explicitly aims to analyse whether EBC in primary care is associated with a reduction in the rate of emergency hospital admissions for ACSC. If the shares of emergency admissions for ACSC are significantly lower in municipalities that established EBC compared to those without such bed capacity, EBC may be a sensible way of redesigning chronic care. Our results support such a view, at least for some diagnoses. We find that EBC is negatively associated with changes in emergency admissions for ACSC. Our rigorous robustness checks also confirm these negative associations with alternative model specifications. However, the disease-specific analyses show that the introduction of an EBC in primary care significantly reduces the rates of emergency department admissions for angina, COPD and asthma only.

The effect of the intervention is analysed with a quasi-experimental design (DID), based on municipalities allocated to a control and intervention group depending on whether or not EBC was established by the end of 2012. However, this identification strategy may raise some concerns. One concern is whether the decision to establish EBC is exogenous. One could imagine that those municipalities that introduce emergency bed capacities early are characterized by limited co-ordination between primary and secondary care in the first place. Limited co-ordination could have led to relatively high numbers of emergency admissions. Thus, the selection of “early birds” is systematically biasing our estimates. However, alternative robustness checks, particularly the one in which we created EBC municipalities arbitrarily using Norwegian county numbers, show that the interaction coefficient for the EBC group of municipalities is insignificant. Furthermore, an alternative post-reform definition gives the same results. Moreover, in all our specifications to capture municipality time-invariant differences, difference over time and differences over time common to all municipalities are modelled using municipality fixed-effects and yearly fixed-effects, respectively. These findings may give some support to our identification strategy.

Secondly, the DID approach relies on the parallel trends assumption. Even if one of the robustness checks support this assumption (see ““Artificial” reform period (i)”), our main concern is that we have only limited data points both before and after reform to test the assumption in a reasonably convincing way.

Finally, an issue we briefly mentioned in the introduction: the Coordination Reform introduced three policy instruments at the same time. Although municipalities could implement EBC “at will” as long as they met the deadline, the interplay with the co-payment scheme and discharge fees in relation to emergency admissions at hospitals are not clear-cut. Nevertheless, Askildsen et al. [2] find no clear indication that co-payment reduced hospital utilization.

Overall, one should be cautious interpreting our findings as causal relations. Thus, the significant associations between EBC and emergency hospital admissions as a basis for policy recommendations must come with reservations. Nonetheless, our results indicate that relatively less costly EBC in primary care can lead to a reduction in aggregate emergency department hospital admissions for a set of ACSC. Emergency department hospital admissions are likely to be costlier in terms of both direct costs (e.g. wages, types of specialist hired) and indirect costs (e.g. drawing resources away from planned admissions) compared to EBC in primary care. Rightly, this assumption relies on EBC being used as envisaged by health authorities: for the less serious cases, freeing hospital capacity to take care of the more serious cases. If this is the case, our findings are only necessary conditions but not sufficient conditions for a net positive welfare effect of the EBC scheme. To the extent that patients with ACSC have at least as good outcomes (e.g. equal or lower readmissions or death rates) in the primary care setting compared to emergency hospital admissions, the potential welfare gains are greater. This could be a topic for future research accounting explicitly for differences in costs between EBC and hospital emergency beds.

## Figures and Tables

**Fig. 6:**
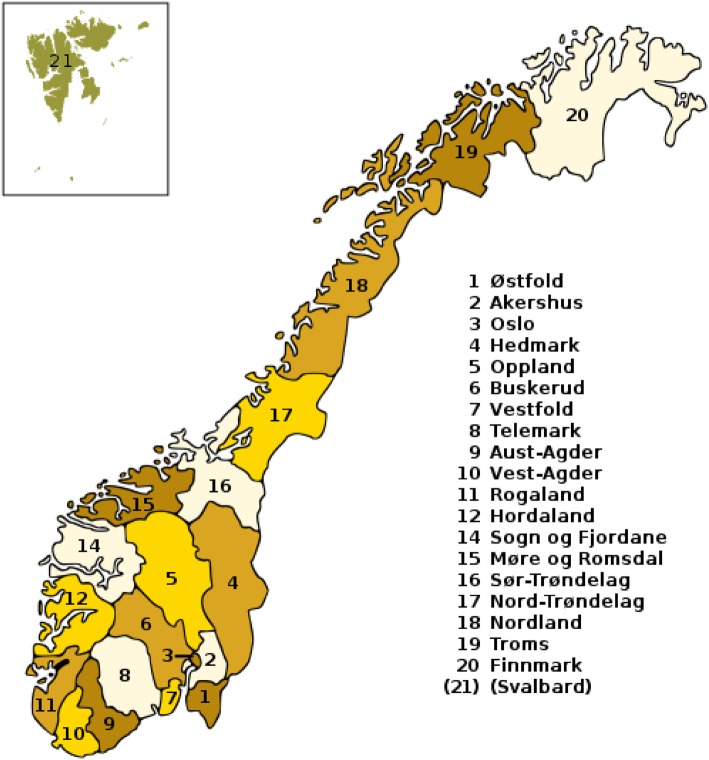
Counties of Norway. (Source: https://en.wikipedia.org/wiki/Counties_of_Norway)

## Appendix

See Fig. 6.

## Footnotes

1 Askildsen et al. [2] concluded that the use of specialist somatic health-care services has not changed as a result of the municipal co-payment system, using data from the same time period as here (2010–2013).

2 About USD 474 or EUR 434.

3 ACSC is customarily grouped in the categories of chronic, acute and vaccine-preventable (see, for example, Tian et al. [20]). See Purdy et al. [13] for a discussion of different definitions of ACSC and the associated disease codes.

4 We omitted 10 municipalities because of ambiguity regarding the timing of the implementation of EBC. These municipalities appear to have introduced EBC before the reform year (2012). See Fig. 1.

5 Model B1: reform × treat(${\hat {\beta }_3}$); model B2: reform1 × treat ($\beta _{3}^{{{\text{B}}2}}$**);** model S1: reform × treat_1_($\beta _{3}^{{{\text{S}}1}}$**)**; model S2: reform_1_ × treat_1_($\beta _{3}^{{{\text{BS}}2}}$**)**.

## References

[1] Angrist JD, Pischke J-S (2009). Mostly Harmless Econometrics: An empiricist's Companion.

[2] Askildsen JE, Holmås TH, Kaarbøe O, Monstad K (2016). Evaluation of health care reform: the introduction of municipal copayment. Tidsskrift for omsorgsforskning, Nr. 2.

[3] Bardsley M, Blunt I, Davies S, Dixon J (2013). Is secondary preventive care improving? Observational study of 10-years trends in emergency admissions for conditions amenable to ambulatory care. BMJ Open.

[4] Blunt I (2013). Focus on preventable admissions. Trends in emergency admissions for ambulatory care sensitive conditions, 2001–2013. Quality Watch.

[5] Gill PJ, Goldacre MJ, Mant D, Heneghan C, Thomosn A, Seagroatt V, Hamden A (2013). Increase in emergency admissions to hospital for children aged under 15 in England, 1999–2010: national database analysis. Arch. Dis. Child..

[6] Ham C (2010). The ten characteristics of the high-performing chronic care system. Health Econ. Policy Law.

[7] Johnson PJ, Ghildayal N, Ward AC, Westgard B, Boland LL, Hokanson JS (2012). Disparities in potentially avoidable emergency department (ED) care. ED visits for ACSC. Med. Care.

[8] Laditka JN, Laditka SB, Mastanduno MP (2003). Hospitalization utilization for ambulatory care sensitive conditions: health outcome disparities associated with race and ethnicity. Soc. Sci. Med..

[9] Lui CK, Wallace SP (2011). A common denominator: calculating hospitalization rates for ACSC in California. Prev. Chronic Dis..

[10] Magán P, Alberquilla Á, Otero Á, Ribera JM (2011). Hospitalizations for ambulatory care sensitive conditions and quality of primary care: their relation with socioeconomic and health care variables in the Madrid Regional Health Service (Spain). Med. Care.

[11] Oster A, Bindman A (2003). Emergency department visits for ambulatory care sensitive conditions. Med. Care.

[12] Probst JC, Laditka JN, Laditka SB (2009). Association between community health center and rural health clinic presence and county-level hospitalization rates for ambulatory care sensitive conditions: an analysis across eight US states. BMC Health Serv. Res..

[13] Purdy S, Griffin T, Salisbury C, Sharp D (2009). Ambulatory care sensitive conditions: terminology and disease coding need to be more specific to aid policy makers and clinicians. Public Health.

[14] Rizza P, Bianco A, Pavia M, Angelillo IF (2007). Preventable hospitalization and access to primary health care in an area of Southern Italy. BMC Health Serv. Res..

[15] Roos LL, Walls R, Uhanova J, Bond R (2005). Physician visits, hospitalization, and socioeconomic status: ambulatory care sensitive conditions in a Canadian setting. Health Serv. Res..

[16] Sanderson C, Dixon J (2000). Conditions for which onset or hospital admission is potentially preventable by timely and effective ambulatory care. J. Health Serv. Res. Policy.

[17] Starfield B (1994). Is primary care essential?. Lancet.

[18] Starfield B (1979). Measuring the attainment of primary care. J. Med. Educ..

[19] Thwaites, R., Glasby, J., Mesurier, N., Littlechild, R.: 2017. Room for one more? A review of the literature on ‘inappropriate’ admissions to hospital for older people in the English NHS. Health Soc. Care Community **25**(1), 1–10 (2017) 10.1111/hsc.12281 26439460

[20] Tian Y, Dixon A, Gao H (2012). Emergency Hospital Admissions for Ambulatory Care-Sensitive Conditions: Identifying the Potential for Reductions. Data Briefing.

[21] van Loenen T, van den Berg MJ, Westert GP, Faber MJ (2014). Organizational aspects of primary care related to avoidable hospitalization: a systematic review. Fam. Pract..

[22] Weeks WB, Ventelou B, Paraponaris A (2016). Rates of admission for ambulatory care sensitive conditions in France in 2009–2010. Trends, geographic variation, costs and an international comparison. Eur. J. Health Econ..

[23] White Paper (2008). White Paper No. 47 (2008–2009).

